# Optic Disc Perfusion in Primary Open Angle and Normal Tension Glaucoma Eyes Using Optical Coherence Tomography-Based Microangiography

**DOI:** 10.1371/journal.pone.0154691

**Published:** 2016-05-05

**Authors:** Karine D. Bojikian, Chieh-Li Chen, Joanne C. Wen, Qinqin Zhang, Chen Xin, Divakar Gupta, Raghu C. Mudumbai, Murray A. Johnstone, Ruikang K. Wang, Philip P. Chen

**Affiliations:** 1 Department of Ophthalmology, University of Washington, Seattle, Washington, United States of America; 2 Department of Bioengineering, University of Washington, Seattle, Washington, United States of America; 3 Department of Ophthalmology, Beijing Anzhen Hospital, Capital Medical University, Beijing, China; Massachusetts Eye & Ear Infirmary, Harvard Medical School, UNITED STATES

## Abstract

**Purpose:**

To investigate optic disc perfusion differences in normal, primary open-angle glaucoma (POAG), and normal tension glaucoma (NTG) eyes using optical microangiography (OMAG) based optical coherence tomography (OCT) angiography technique.

**Design:**

Cross-sectional, observational study.

**Subjects:**

Twenty-eight normal, 30 POAG, and 31 NTG subjects.

**Methods:**

One eye from each subject was scanned with a 68 kHz Cirrus HD-OCT 5,000-based OMAG prototype system centered at the optic nerve head (ONH) (Carl Zeiss Meditec Inc, Dublin, CA). Microvascular images were generated from the OMAG dataset by detecting the differences in OCT signal between consecutive B-scans. The pre-laminar layer (preLC) was isolated by a semi-automatic segmentation program.

**Main Outcome Measures:**

Optic disc perfusion, quantified as flux, vessel area density, and normalized flux (flux normalized by the vessel area) within the ONH.

**Results:**

Glaucomatous eyes had significantly lower optic disc perfusion in preLC in all three perfusion metrics (p<0.0001) compared to normal eyes. The visual field (VF) mean deviation (MD) and pattern standard deviation (PSD) were similar between the POAG and NTG groups, and no differences in optic disc perfusion were observed between POAG and NTG. Univariate analysis revealed significant correlation between optic disc perfusion and VF MD, VF PSD, and rim area in both POAG and NTG groups (p≤0.0288). However, normalized optic disc perfusion was correlated with some structural measures (retinal nerve fiber layer thickness and ONH cup/disc ratio) only in POAG eyes.

**Conclusions:**

Optic disc perfusion detected with OMAG was significantly reduced in POAG and NTG groups compared to normal controls, but no difference was seen between POAG and NTG groups with similar levels of VF damage. Disc perfusion was significantly correlated with VF MD, VF PSD, and rim area in glaucomatous eyes. Vascular changes at the optic disc as measured using OMAG may provide useful information for diagnosis and monitoring of glaucoma.

## Introduction

Glaucoma is the leading cause of irreversible blindness worldwide [1], and elevated intraocular pressure (IOP) remains a major risk factor for its development and progression [2,3]. Primary open-angle glaucoma (POAG) and normal tension glaucoma (NTG) have typically been separated based on IOP, with POAG patients having a history of IOP higher than 21 mmHg, and NTG patients having an untreated IOP that is consistently lower than 21 mmHg; some authors have found NTG comprises up to two-thirds of all glaucoma patients [4,5]. Recent studies have proposed that POAG and NTG may represent a continuum of open-angle glaucoma that differ primarily in predominant causative risk factors, with higher IOP being important in POAG, while additional IOP-independent factors may be important in NTG [6–8].

Vascular dysfunction in the optic nerve head (ONH) has been proposed as a contributing factor in the development and progression of glaucoma, especially in NTG patients [9]. This is supported by the increased frequency of optic disc hemorrhage, migraine headaches, Raynaud’s phenomenon, and primary vascular dysregulation syndrome (Flammer syndrome) in NTG patients compared to POAG patients [7, 10–13]. Several reports have measured directly or have calculated indirectly the ONH blood flow in vivo, using techniques such as intravenous fluorescein angiography (FA), mean ocular perfusion pressure (MOPP), nocturnal hypotension, and C-reactive protein levels, but the results so far are inconsistent [7,14–19].

We previously described the optical microangiography (OMAG) imaging technique [20] for visualization of vascular function in the retina and ONH [21,22], based on Fourier-domain optical coherence tomography (FD-OCT). Optical microangiography generates three-dimensional, microscopic resolution structural images as well as vascular network images in a non-contact, non-invasive fashion, by detecting the differences in the scattered light caused by the moving particles, for example, red blood cells in the vessels, between consecutive B-scans at the same transversal location in the ONH over time, allowing visualization and measurement of perfusion.

The purpose of this study was to use OMAG to visualize, quantify, and compare ONH perfusion in normal, POAG and NTG eyes.

## Methods

This study was approved by the Institutional Review Board of the University of Washington (UW) and written informed consent was obtained from all subjects before imaging. This study followed the tenets of the Declaration of Helsinki and was conducted in compliance with the Health Insurance Portability and Accountability Act.

Subjects with POAG, NTG or with normal optic discs were prospectively enrolled at the UW Eye Institute. Inclusion criteria were best-corrected visual acuity of 20/40 or better, refractive error between -6 and +3 D spherical equivalent, no media opacities that prevented high-quality imaging of the optic disc, and for the normal group, a normal retinal nerve fiber layer (RNFL) thickness on FD-OCT. Exclusion criteria were previous diagnosis of migraine, use of medications known to affect the retina, any ocular disease other than glaucoma or cataract, and previous intraocular surgeries other than uncomplicated glaucoma or cataract surgery.

The diagnosis of glaucoma was based on the presence of characteristic glaucomatous optic neuropathy, irrespective of visual field loss; POAG eyes had a history of IOP > 21 mmHg, and NTG eyes had no known IOP > 21 mmHg. All subjects underwent a comprehensive ophthalmologic examination at time of enrollment, and glaucoma subjects received a visual field (VF) exam to determine mean deviation (MD) and pattern standard deviation (PSD). All VF tests were performed on Humphrey Field Analyser II (Carl Zeiss Meditec, Dublin, CA), and only reliable tests were included (≤33% fixation losses, false-negative results and false-positive results). One eye from each subject was included in this study. A single eye was randomly selected and imaged if both were eligible.

We collected blood pressure (BP) measurements for a subgroup of subjects that were followed at UW for other medical conditions to determine if differences in calculated MOPP were present. Mean ocular perfusion pressure was defined as 2/3(mean arterial pressure—IOP), where mean arterial pressure = diastolic BP + 1/3(systolic BP–diastolic BP) [23]. All BP measurements were taken within one year before or after the OMAG scan. None of the included subjects had changes in blood pressure medications during the time period reviewed.

**Fig 1:**
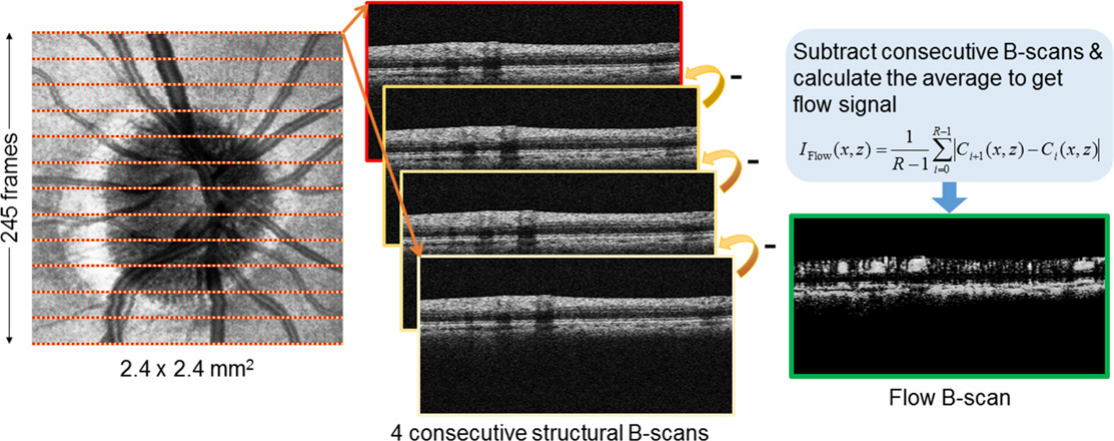
Example showing how flow signal is detected by optical microangiography (OMAG) algorithm. Four consecutive B-scans are acquired at each fixed transversal location on the optic disc. The final blood flow signal is calculated by subtracting consecutive B-scan pairs, and calculating the average of differences at each location.

All eyes were scanned using a 68 kHz Cirrus 5000 HD-OCT based OMAG prototype system (center wavelength at 840 nm) with active motion-tracking capability (Carl Zeiss Meditec Inc, Dublin, CA). The active motion tracking, a unique feature in the system, provides almost motion-free blood flow images, which is important for quantitative assessment of the flow metrics described below [24]. Three OMAG scans centered on the ONH were acquired. The OMAG scan pattern generated a volumetric dataset over a 2.4×2.4 mm^2^ area centered on the optic disc. Four consecutive B-scans were acquired at each fixed transversal (i.e. along the fast axis) location before the scanning probe moved to the next transversal location on the optic disc (Fig 1). A total of 245 transversal locations were sampled. The time difference between two successive B-scans was approximately 3.6 msec.

In addition, an Optic Disc Cube 200x200 raster cube OCT scan was acquired using the same prototype device at the same visit to obtain global average RNFL thickness and ONH structural measurements. The scan provided coverage of 6×6 mm^2^ centered on the optic disc. The OMAG scans and the structural raster cube were considered poor quality images and omitted from further analysis if the signal strength fell below the manufacturer recommended cutoff (<6) or if they showed noticeable eye movement.

All the acquired volumetric scans were processed with an OMAG algorithm to extract both the structural and blood flow signals. The details of the OMAG algorithm have been described elsewhere [20,21–25]. In brief, the basic idea of OMAG algorithm is to differentiate the backscattered signals created by the moving particles (red blood cells) from those created by the static retinal tissues. Since the signal from static retinal tissue keeps steady, while the signal from vessels changes over time, blood flow signals could be calculated by subtracting complex OCT signals between each consecutive B-scans pair at each transversal location (Fig 1) [20,21]. The intensity of the blood flow signal was proportional to the amount of red blood cells (RBCs) passing through the vessels [26,27]. Finally, the average of the differences at each location was calculated to generate a final blood flow signal, *I*_Flow_(*x*, *z*), as described in Eq (1):

$$I_{\text{Flow}}\left(x,z\right)=\frac{1}{R-1}\sum_{i=0}^{R-1}|C_{i+1}\left(x,z\right)-C_{i}\left(x,z\right)|,$$ (1)

where *i* is the index of the repeated time of B-scans at each transversal location, *C*(*x*, *z*) indicates the complex OCT signal at *x*th A-scan and *z*th sampling point in the axial direction. *R*(= 4) is the number of repeated B-scans.

**Fig 2:**
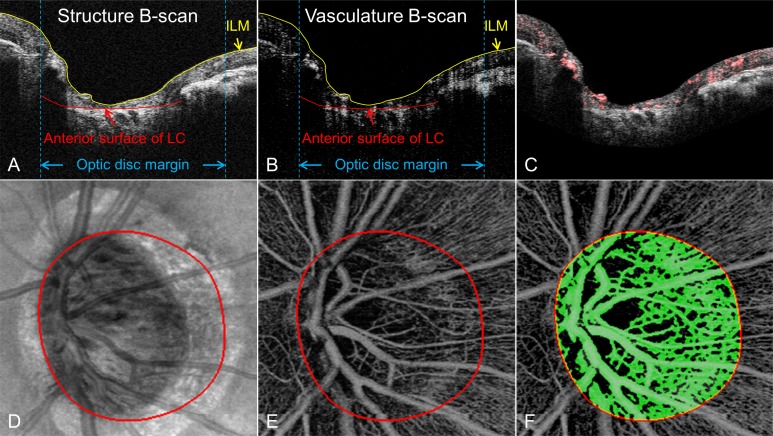
Example of retinal layer segmentation and structural and vascular *en face* images of a glaucomatous eye. **(A)** and **(B)**: structural and vasculature cross-sectional image superimposed with segmented retinal boundaries. Two retinal boundaries were segmented: inner limiting membrane (ILM) (the yellow line), retinal pigment epithelium (RPE) (the red line outside the optic disc) and anterior surface of lamina cribrosa (LC) (the same red line within the optic disc). **(C)** presents a structural cross-sectional superimposed with detected blood flow signal (in red). **(D)** shows the structural *en face* images, **(E)** and **(F)** present the vascular *en face* images and the detected blood vessel map from pre-laminar tissue.

Our OMAG prototype device is equipped with an 840 nm laser source, resulting in limited light penetration into deep tissues, including the lamina cribrosa tissue of the optic nerve. Therefore, we focused on the pre-laminar tissue (preLC) only. Based on the structural images, the boundaries of the inner limiting membrane (ILM) and the anterior surface of the lamina cribrosa were delineated using a semi-automatic retinal layer segmentation program to isolate the preLC (Fig 2A) [28]. The segmentation algorithm automatically detected the boundaries of the retinal layers from the structural OCT cross-sectional images by measuring the gradient of OCT signals [28]. If the boundaries were insufficiently clear for automated detection, the operator could manually guide the algorithm to identify the correct boundaries. Since the vascular images were derived from the structural images, the same boundaries were then applied to OMAG vascular images (Fig 2B). Maximum intensity projection analyses, which detected the blood flow signal with highest intensity value along each A-scan, were performed within the segmented retinal layer to generate vascular *en face* images in the preLC layer (Fig 2E). The optic disc margin was manually delineated and defined by identifying the end of Bruch’s membrane on structural B-scans.

To quantify optic disc perfusion, we created three quantitative measurements: flux, vessel area density, and normalized flux [29]. The flux was defined as the number of particles passing through the cross-section per unit area as shown in Eq (2):

$$\text{Flux}=\frac{\sum_{\left(x,y\right)\in \text{ONH}}\frac{I_{\text{Flow}}\left(x,y\right)}{255}}{Area_{\text{ONH}}},$$ (2)

where the blood flow signal *I*_Flow_ was normalized to between 0 and 1 by dividing by 255 (the full dynamic range of the flow signal), and thus represented a ratio without a unit.

Vessel area density was the ratio of vessel area within the ONH to the area of the ONH. Blood vessels in the ONH were detected by a multiscale Hessian filter, developed based on the method of Frangi et al [30], from the vascular *en face* images. The Hessian filter measured the curvature of blood vessels in a small region of the vascular *en face* images, and this structural information was converted into probability-like estimates of vesselness (Fig 2F).

Given that the amount of viable tissue determines optic disc perfusion, in order to avoid bias from a reduced amount of ONH tissue in glaucomatous eyes, we measured the normalized flux, which was the flux normalized by the vessel area, to evaluate the flux in the vessels only. Similar to the flux, the blood flow signal was normalized to between 0 and 1 by dividing by the full dynamic range of blood flow signal intensity, and thus represented a ratio without a unit.

Preliminary data from prior studies (unpublished data) was used to guide sample size calculation. Given a mean normalized flux in POAG patients of 0.300 and SD of 0.06, the sample size needed to detect a 15% difference in normalized flux between glaucoma groups was calculated as 28 per group, with 80% power and alpha error set to 0.05. ANOVA was used to compare baseline demographic information, ONH structural biometric measurements, and preLC optic disc perfusion metrics among normal, POAG and NTG eyes. Two-tailed, independent samples t-tests were used for the comparisons between POAG and NTG eyes. Univariate linear regression models were used to investigate the correlation between disc perfusion metrics and other clinical assessment measurements. All statistical analyses were performed using JMP® Pro 12.1.0 (SAS Institute Inc., Cary, NC). P<0.05 was considered statistically significant.

## Results

Thirty eyes from 30 normal subjects, 33 eyes from 33 POAG subjects, and 33 eyes from 33 NTG subjects were enrolled. Two normal, three POAG and 2 NTG eyes were excluded because of poor signal quality and/or eye movement. Table 1 summarizes the demographic information and structural biometric measurements. No significant differences were detected in age, gender, systolic and diastolic blood pressure, and MOPP (p≥0.20). Intraocular pressure was significantly lower in the NTG group compared to POAG group (p = 0.0139). Normal eyes showed significantly higher global average RNFL thickness and rim area and significantly lower averaged cup/disc ratio compared to POAG and NTG eyes (all p<0.0001).

**Table 1:** Baseline demographic information and optic nerve head structural biometric measurements.

	Normal (N = 28)	POAG (N = 30)	NTG (N = 31)	P-value
Age (yrs)*	68.6 ± 10.4 (64.4, 72.9)	64.6 ± 10.9 (60.5, 68.6)	66.2 ± 12.2 (62.2, 70.2)	0.38
Intraocular Pressure (mmHg)*	13.5 ± 3.4 (12.1, 14.9)	14.8 ± 4.0 (13.5, 16.1)	12.0 ± 3.3 (10.7, 13.3)	**0.0139**
**Male / Female**	16 / 12	16 / 14	12 /19	0.32
Systolic Blood Pressure (mmHg)* ‡	134.7 ± 20.5 (126.7, 142.8)	125.8 ± 19.0 (116.9, 134.7)	132.8 ± 15.6 (123.1, 142.6)	0.31
Diastolic Blood Pressure (mmHg)* ‡	80.4 ± 12.5 (75.4, 85.4)	78.2 ± 12.2 (72.1, 83.7)	74.7 ± 9.6 (68.6, 80.7)	0.35
MOPP (mmHg)*‡	52.1 ± 8.2 (48.6, 55.6)	47.5 ± 9.3 (43.6, 51.3)	51.1 ± 6.6 (46.9, 55.3)	0.20
Global Average RNFL Thickness (μm)*	90.0 ± 11.5 (85.9, 94.2)	64.9 ± 9.3 (60.9, 68.9)	71.6 ± 12.2(67.6, 75.5)	**<0.0001**
Averaged Cup-to-disc Ratio*	0.44 ± 0.17 (0.39, 0.48)	0.74 ± 0.10 (0.70, 0.79)	0.73 ± 0.09 (0.69, 0.78)	**<0.0001**
**Rim Area (mm**^**2**^)*	1.28 ± 0.20 (1.21, 1.35)	0.76 ± 0.19 (0.69, 0.83)	0.80 ± 0.18 (0.74, 0.87)	**<0.0001**

*Mean ± standard deviation with 95% confidence intervals are shown in the parentheses. All comparisons made using ANOVA.

‡ N = 22, 18 and 15 for normal, POAG, and NTG groups, respectively.

Fig (2A–2F) presents exemplar results of the retinal segmentation layer and structural and vascular *en face* images of a glaucomatous eye. Compared with normal eyes, POAG eyes had a significantly lower flux, lower vessel area density, and lower normalized flux in preLC (p<0.0001). Additionally, NTG eyes also had a significantly lower flux, lower vessel area density, and lower normalized flux in preLC when compared with normal eyes (p<0.0001, Table 2).

**Table 2:** Summary of flux, vessel area density, and normalized flux between normal, primary open angle glaucoma (POAG) and normal tension glaucoma (NTG) groups.

Pre-Laminar Tissue	Normal (N = 28)	POAG (N = 30)	NTG (N = 31)	P-value
**Flux**	0.33 ± 0.04 (0.31, 0.35)	0.24 ± 0.07 (0.22, 0.26)	0.25 ± 0.06 (0.23, 0.27)	**<0.0001**
**Vessel Area Density**	0.77 ± 0.02 (0.74, 0.79)	0.68 ± 0.08 (0.66, 0.71)	0.70 ± 0.07 (0.68, 0.73)	**<0.0001**
**Normalized Flux**	0.37 ± 0.04 (0.35, 0.38)	0.29 ± 0.05 (0.27, 0.31)	0.30 ± 0.05 (0.28, 0.32)	**<0.0001**

Mean ± standard deviation with 95% confidence intervals are shown in the parentheses. All comparisons made using ANOVA.

The average MD and PSD did not differ significantly between the POAG and NTG groups (p≥0.31) (Table 3). One eye in the POAG group (3.33%) had undergone trabeculectomy18 months prior to the OMAG scanning. No significant differences were detected in age, gender, systolic and diastolic BP, MOPP, number of IOP lowering medications, corneal pachymetry, cup/disc ratio, and rim area (p≥0.25). Intraocular pressure was significantly lower (p = 0.0048), and global average RNFL thickness was significantly thicker (p = 0.0201) in the NTG group. Comparison of the POAG and NTG groups revealed no significant differences in flux, vessel area density, and normalized flux (p≥0.32, Table 4).

**Table 3:** Baseline demographic information and optic nerve head structural biometric measurements for primary open angle glaucoma (POAG) and normal tension glaucoma (NTG) groups.

	POAG (N = 30)	NTG (N = 31)	P-value
Age (yrs)*	64.6 ± 10.9 (60.3, 68.8)	66.2 ± 12.2 (62.0, 70.3)	0.59
Intraocular Pressure (mmHg)*	14.8 ± 4.0 (13.4, 16.1)	12.0 ± 3.3 (10.7, 13.3)	**0.0048**
**Male / Female**	16 / 14	12 /19	0.25
**Taking Glaucoma Drops, n (%)**	26 (86.7%)	26 (83.9%)	0.76
Number of Glaucoma Drops*	2.0 ± 1.2	1.9 ± 1.4	0.70
**Prostagladin, n (%)**	25 (83.4%)	24 (77.4%)	0.56
**Beta-blocker, n (%)**	17 (56.7%)	17 (54.8%)	0.89
**CAI, n (%)**	10 (33.3%)	9 (29.0%)	0.72
**Alpha-2 agonist, n (%)**	10 (33.3%)	10 (32.2%)	0.93
MOPP* ‡	47.5 ± 9.3 (43.5, 51.4)	51.1 ± 6.6 (46.8, 55.4)	0.21
Corneal Pachymetry*	545.6 ± 39.4 (531.4, 559.8)	534.2 ± 35.6 (519.7, 548.7)	0.26
VF MD*	-5.49 ± 6.79 (-7.67, -3.31)	-4.79 ± 5.04 (-6.93, -2.65)	0.65
VF PSD*	5.82 ± 4.05 (4.30, 7.35)	6.93 ± 4.30 (5.43, 8.43)	0.31
Global Average RNFL Thickness (μm)*	64.9 ± 9.3 (61.0, 68.9)	71.6 ± 12.2 (67.7, 75.5)	**0.0201**
Averaged Cup-to-disc Ratio*	0.74 ± 0.10 (0.71, 0.78)	0.73 ± 0.09 (0.70, 0.77)	0.70
**Rim Area (mm**^**2**^)*	0.76 ± 0.19 (0.69, 0.82)	0.80 ± 0.18 (0.74, 0.87)	0.32

*Data are presented as mean ± standard deviation with 95% confidence intervals shown in the parentheses. All comparisons made using independent samples, 2-tailed t-test. ‡ N = 18 and 15 for POAG, and NTG groups, respectively. CAI = Carbonic anhydrase inhibitor

**Table 4:** Summary of flux, vessel area density, and normalized flux between primary open angle glaucoma (POAG) and normal tension glaucoma (NTG) groups.

Pre-Laminar Tissue	POAG (N = 30)	NTG (N = 31)	P-value
**Flux**	0.24 ± 0.07 (0.21, 0.26)	0.25 ± 0.06 (0.23, 0.27)	0.41
**Vessel Area Density**	0.68 ± 0.08 (0.65, 0.71)	0.70 ± 0.07 (0.67, 0.73)	0.32
**Normalized Flux**	0.29 ± 0.05 (0.27, 0.31)	0.30 ± 0.05 (0.28, 0.32)	0.48

Mean ± standard deviation with 95% confidence intervals are shown in the parentheses. All comparisons made using independent samples, 2-tailed t-test.

**Fig 3:**
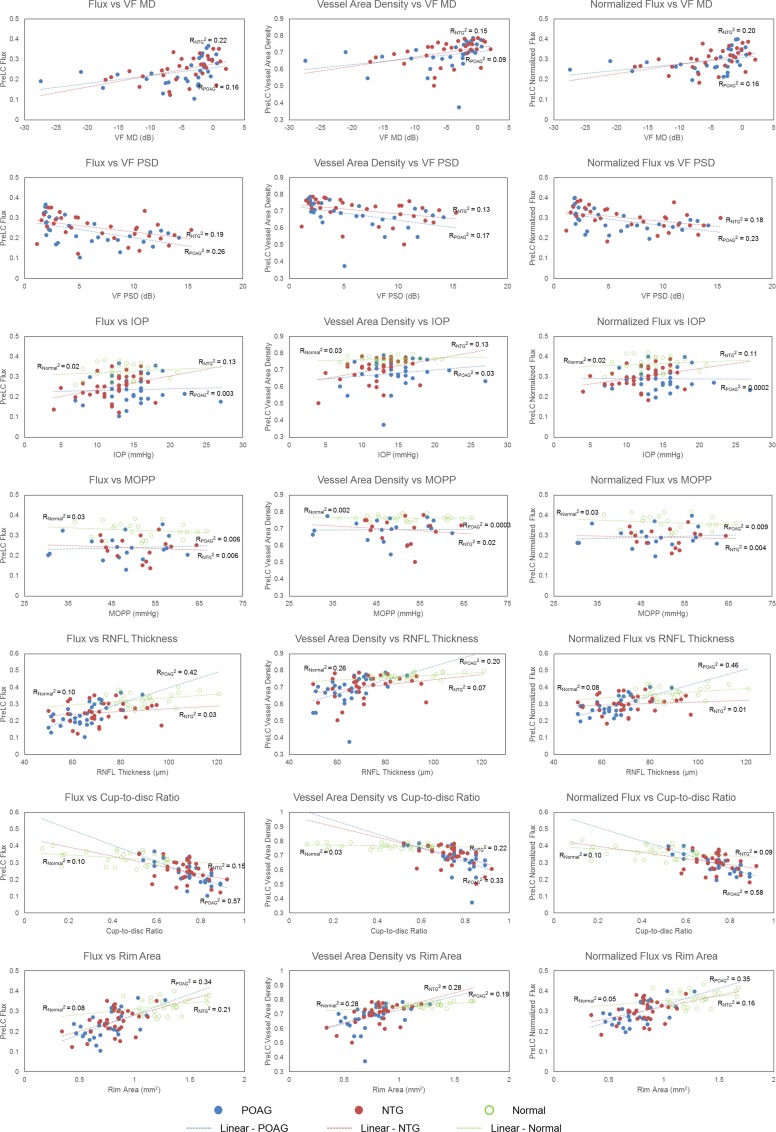
Scatter plots of three groups for three optic disc perfusion metrics and clinical measurements [visual field (mean deviation (MD) and pattern standard deviation (PSD)] and structural biometrics [retinal nerve fiber layer (RNFL) thickness, cup/disc ratio (CDR), and rim area]. R^2^ with p-values in the parentheses are presented (R^2^_N_, R^2^_POAG_, and R^2^_NTG_ indicate R^2^ value for normal, primary open-angle glaucoma, and normal tension glaucoma eyes, respectively).

In the POAG group, univariate linear regression analyses indicated that flux and normalized flux were significantly correlated with VF MD and PSD, global average RNFL thickness, and ONH cup/disc ratio and rim area (p≤0.0303), For the NTG group, flux and vessel area density showed a significant correlation with VF MD and PSD, IOP, and ONH cup/disc ratio and rim area (p≤0.0492, Table 5, Fig 3). Notably, normalized flux in NTG was correlated with VF MD and PSD, and ONH rim area, but not with global average RNFL thickness, nor ONH cup/disc ratio.

**Table 5:** Summary of univariate regression analyses between optic disc perfusion measurement metrics and other functional and structural clinical measurements, for primary open angle glaucoma (POAG),normal tension glaucoma (NTG), and normal groups.

**POAG**			
**Variables**	**Flux**	**Vessel Area Density**	**Normalized Flux**
**VF MD**	0.16 **(0.0275)**	0.09 (0.10)	0.16 **(0.0303)**
**VF PSD**	0.26 **(0.0043)**	0.17 **(0.0258)**	0.23 **(0.0068)**
**IOP**	0.003 (0.79)	0.03 (0.37)	0.0002 (0.94)
**MOPP**	0.006 (0.76)	0.0003 (0.94)	0.009 (0.70)
**Global Average RNFL Thickness**	0.42 **(<0.0001)**	0.20 **(0.013)**	0.46 **(<0.0001)**
**Cup-to-disc Ratio**	0.57 **(<0.0001)**	0.33 **(0.0009)**	0.58 **(<0.0001)**
**Rim Area**	0.34 **(0.0007)**	0.19 **(0.0161)**	0.35 **(0.0005)**
**NTG**			
**Variables**	**Flux**	**Vessel Area Density**	**Normalized Flux**
**VF MD**	0.22 **(0.0082)**	0.15 **(0.0288)**	0.20 **(0.0111)**
**VF PSD**	0.19 **(0.0139)**	0.13 **(0.0492)**	0.18 **(0.018)**
**IOP**	0.13 **(0.0441)**	0.13 **(0.0433)**	0.11 (0.07)
**MOPP**	0.006 (0.78)	0.02 (0.65)	0.004 (0.82)
**Global Average RNFL Thickness**	0.03 (0.38)	0.07 (0.15)	0.01 (0.54)
**Cup-to-disc Ratio**	0.15 **(0.0332)**	0.22 **(0.0082)**	0.09 (0.09)
**Rim Area**	0.21 **(0.0094)**	0.28 **(0.002)**	0.16 **(0.028)**
**Normal**			
**Variables**	**Flux**	**Vessel Area Density**	**Normalized Flux**
**IOP**	0.02 (0.48)	0.03 (0.40)	0.02 (0.52)
**MOPP**	0.03 (0.45)	0.002 (0.86)	0.03 (0.44)
**Global Average RNFL Thickness**	0.10 (0.11)	0.26 **(0.006)**	0.08 (0.13)
**Cup-to-disc Ratio**	0.10 (0.09)	0.03 (0.35)	0.10 (0.10)
**Rim Area**	0.08 (0.16)	0.28 **(0.0041)**	0.05 (0.23)

Data are presented as R^2^ with p-values in the parentheses.

## Discussion

In the present study, we investigated ONH perfusion among POAG, NTG, and normal eyes using OMAG. Significant differences in blood flux within the preLC were found between normal and glaucomatous eyes, but not between POAG and NTG eyes in which VF MD and PSD were similar. Reduction in optic disc perfusion was significantly correlated with worse VF parameters and thinner rim area in both POAG and NTG groups. However, normalized optic disc perfusion was correlated with some structural measures in POAG but not NTG eyes (global average RNFL thickness and ONH cup/disc ratio)

In OMAG, when the incident light is backscattered by retinal tissue, the OCT signal remains steady, but it changes over time when the light is backscattered by moving RBCs. Based on this concept, OMAG generates ONH microcirculatory information by subtracting two consecutive B-scans. In doing so, the OCT signals from static retinal tissues are removed while the signals from moving blood cells remain. By averaging differences of each repetition, OMAG further enhances the signal-to-noise ratio and generates blood flow signals with better contrast. Since we were detecting the blood flow signals caused by the moving blood cells and then projecting 3D blood flow signals onto 2D vascular *en face* images, the optic disc perfusion measured here is considered as the flux—number of RBCs crossing through per unit area—of the RBCs, which has been shown to be correlated with blood flow in vivo [26,27].

Vascular dysfunction has been proposed to play an important role for the development and progression of glaucomatous optic neuropathy; some previous studies have proposed that this role is more important in NTG than in POAG eyes [9,31]. However, other studies have not found significant differences in ONH perfusion between POAG and NTG, using a variety of imaging techniques. Hitchings et al. used FA to compare papillary and peripapillary circulation between POAG and NTG with similar functional and structural damage, and found no evidence of difference in circulation times between the 2 groups [32]. Hamard et al. compared the velocity of red blood cells in ONH capillaries, and found it was similarly reduced in both POAG and NTG groups compared to normal controls, using laser Doppler flowmetry (LDF) [33]. Color Doppler imaging studies have found reduced blood flow velocity in glaucomatous eyes compared to normal eyes, but no difference between POAG and NTG groups [34,35]. Yaoeda et al. studied the relationship between ONH blood flow velocity and VF loss in patients with POAG and NTG, and found no significant differences between POAG and NTG groups, using laser speckle flowgraphy (LSFG) [23].

Our results using OMAG blood flow measurements are mostly in agreement with these prior findings. However, OMAG has advantages over other forms of vascular imaging of the ONH. Fluorescein angiography requires intravenous injection of contrast [36], and only provides information on the most superficial vasculature of the ONH; blood flow in deeper layers can be analyzed qualitatively only by evaluating the density of the diffused staining. Color Doppler imaging only measures the retrobulbar circulation; LDF can only examine a small part of the ONH, with the location of the volume included in the measurement remaining undefined [9]; and LSFG measures blood flow velocity, but can only assess the accumulated speckle changes in two-dimensions. The OMAG method is non-invasive, can evaluate blood flow in deeper portions of the ONH and over the entirety of the ONH, in three dimensions. We have also found OMAG to have high intra- and inter-observer repeatability and reproducibility (coefficient of variation ≤ 3.7% (intra-observer) and intraclass correlation coefficient ≥ 0.987 (inter-observer); unpublished data), which compares favorably to traditional methods, such as Doppler optical coherence tomography [37].

In our study, univariate regression analyses between blood flow metrics and functional and structural measurements revealed significant correlation between flux and normalized flux and VF parameters in both POAG and NTG groups, and significant correlations between flux and normalized flux and all structural biometrics in POAG, while in the NTG group, flux and normalized flux were significantly correlated with rim area. Some authors have shown that VF defects in NTG patients typically appear deeper, steeper and closer to fixation than in POAG patients [38,39], but others have found no difference between them [40,41]. In addition, some authors have found that NTG patients tend to have more localized defects of the RNFL, and deeper, focal notching of the rim [42–44], but others have found that the optic disc appearance was similar between the two groups [40,41,45]. Our results support that similar vascular changes are seen in similar levels of functional damage in POAG and NTG, but differences in structural findings such as global average RNFL thickness and ONH cupping may exist between POAG and NTG, given that normalized flux in NTG did not correlate with those structural measurements, and this may imply that pathophysiologic differences may exist between POAG and NTG. For example, a given level of diffuse RNFL damage and generalized increase in cup/disc ratio in POAG may result in a similar level of overall optic disc perfusion as a more dense, focal defect of the same structural measures in NTG, though differing levels of RNFL thinning and ONH cupping may be seen between the two groups.

Our study has several limitations. It remains unclear if the vascular changes that we detected are the cause or the result of glaucomatous ONH damage; only prospective longitudinal studies can determine that. Previous studies have shown that IOP lowering medications may increase blood flow in the ONH [46, 47]. Most of our glaucoma subjects were taking antihypertensive eye drops, though no significant difference in the number or type of medications was detected between POAG and NTG groups (p≥0.56). In most (88%) of our patients, BP measurements were not taken at the time of the OMAG scan, and MOPP was based on retrospective BP measurements. However, a previous study using OCT angiography with contemporaneously collected BP data also found no significant correlations between MOPP and optic disc perfusion [48]. We did not obtain visual field testing in our normal subjects, though all normal subjects underwent comprehensive ocular examination and had statistically normal global average peripapillary RNFL thickness and normal optic disc measures using FD-OCT. We did not attempt to segment different sectors of the ONH to determine if such measurements might explain some of the differences in correlations between POAG and NTG eyes. Lastly, some symptoms of systemic vascular dysregulation may occur only episodically (e.g., after cold exposure or at night), and it is possible that provocative tests may be needed to uncover differences in perfusion between POAG and NTG.

In conclusion, ONH perfusion metrics detected by OMAG were significantly lower in glaucomatous than normal eyes within the preLC, and POAG and NTG exhibited similar reductions in ONH perfusion. Further prospective studies may reveal if vascular dysfunction is a risk factor for the development and progression of POAG and/or NTG.
